# AI enabled decision support systems in epilepsy surgery a scoping review

**DOI:** 10.21203/rs.3.rs-8612799/v1

**Published:** 2026-02-19

**Authors:** Kai Yu, Shuang Zhou, Meijia Song, Zaifu Zhan, Yu Hou, Yiran Song, Min Zeng, Biao Yin, Feifan Liu, Sandipan Pati, Zhiyi Sha, Mingquan Lin, Rui Zhang

**Affiliations:** University of Minnesota; University of Minnesota; University of Minnesota; University of Minnesota; University of Minnesota; University of Minnesota; University of Minnesota; University of Massachusetts Chan Medical School; University of Massachusetts Chan Medical School; University of Minnesota; University of Minnesota; University of Minnesota; University of Minnesota

## Abstract

Artificial intelligence is increasingly explored to support decision-making in epilepsy surgery, yet evidence for implementation across the epilepsy surgery pathway remains limited. We conducted a scoping review of 145 studies published between January 2018 and May 2025 to map AI-enabled decision support systems across surgical stages and clinical tasks, characterize datasets by modality, size, geographic provenance and accessibility, and synthesize modeling practices, external validation and workflow integration. The literature is heavily concentrated in the pre-operative stage, with no included intra-operative studies and relatively few post-operative applications. Most studies rely on small, single-center and non-public datasets and use supervised CNN-based models. External validation and workflow-integrated evaluation are uncommon, and only a minority of systems report semi-integrated clinical workflows. These findings highlight key gaps in generalizability, workflow readiness and equity, and inform priorities for multi-center data resources, rigorous cross-site evaluation and clinically meaningful endpoints to enable safe, scalable adoption.

## Introduction

Epilepsy surgery is a high-risk, resource-intensive, multidisciplinary care pathway that requires coordinated, time-sensitive decisions across pre-operative, intra-operative, and post-operative stages. Epilepsy affects more than 50 million people worldwide^1^, and global epidemiological estimates suggest that approximately 10.1 million people living with epilepsy are potential surgical candidates, with around 1.4 million new surgically treatable cases each year^2^. Despite its promise for drug-resistant epilepsy, surgical success depends on integrated presurgical evaluation, precise operative execution, and careful post-operative monitoring to maximize seizure control while minimizing the risk of neurological complications^3–5^. Because workflows, expertise, and data infrastructures vary across centers, achieving consistent decision-making and scalable implementation remains a health-system challenge.

Across the pre-operative, intra-operative, and post-operative phases of epilepsy surgery, clinicians rely on a wide range of diagnostic modalities, including magnetic resonance imaging (MRI), positron emission tomography (PET), computed tomography (CT), scalp electroencephalography (EEG), and intracranial EEG, to support multidisciplinary decisions such as lesion detection, seizure-onset (SOZ) and epileptogenic zone (EZ) localization, electrode navigation, and postoperative outcome assessment. However, these decisions rarely rely on any single modality. Instead, they depend on how multimodal data are synthesized and interpreted, a process that remains inherently complex and variable. Interpretation is often influenced by center-specific practices, and clinician experience, leading to substantial inter-institution variability even when similar diagnostic modalities are available. This variability complicates standardization and limits the scalability of consistent decision support across epilepsy centers.

Beyond interpretive subjectivity, epilepsy surgery workflows are further challenged by the substantial specialist time and expertise required to interrogate complex diagnostic data. Depending on the clinical question, clinicians may need to review hours to weeks of scalp or intracranial EEG recordings, scrutinize high-resolution structural MRI for subtle cortical abnormalities, interpret regional metabolic patterns on PET, or evaluate post-operative structural changes on CT. These datasets are high-dimensional, noisy, acquired under heterogeneous protocols, and demand specialized expertise that varies across clinicians and centers. As a result, manual analysis is not only time-intensive but also vulnerable to inter-observer variability, limiting standardization and scalability in surgical decision-making. These constraints motivate workflow-integrated, scalable decision-support systems that can distill clinically meaningful features and enable more reproducible evaluation across all stages of epilepsy surgery.

With the rapid development of artificial intelligence (AI), an increasing number of studies have explored AI-enabled decision support across the epilepsy surgery pathway. Most of this literature is based on deep learning methods. These approaches have shown promise in tasks such as structural MRI–based lesion detection^6–8^, intracranial EEG–based SOZ/EZ localization^9–11^, automated functional mapping^12,13^, surgical planning and navigation^14–16^, and surgical outcomes prediction^17–20^. A small number of studies have also begun to investigate foundation model approaches, for example large language models (LLMs) for clinical text interpretation^21^. However, the literature remains largely focused on technical feasibility in controlled settings. Many systems remain offline research prototypes, some are developed as standalone decision-support tools^22,23^, and a small number have been partially integrated into existing clinical workflows^24^. Health systems still lack the evidence needed to decide whether and how these tools can be deployed. Cross-center validation and evaluations of workflow integration, patient impact, and safety are still uncommon.

Current research on AI-enabled decision support in epilepsy surgery remains fragmented across clinical tasks, data modalities, and study designs, making it difficult to judge where evidence is mature enough for implementation across the full care pathway. Existing review articles have largely reflected this fragmentation. Many focus on a single component of the surgical workflow or on a single modality, which limits the ability of clinicians and health systems to compare approaches, prioritize prospective evaluation, and plan workflow integration. For example, several reviews concentrate on EEG or intracranial EEG analysis^25–30^, providing insights into electrophysiology-based SOZ/EZ localization but offering limited discussion of imaging-based approaches or post-operative evaluation. Conversely, neuroimaging-focused reviews centered on MRI, PET, or CT often exclude electrophysiological studies entirely, resulting in an incomplete and modality-isolated picture of current evidence^31–33^. This separation across modalities and tasks prevents a pathway level assessment of AI systems, including how they are validated, evaluated for clinical impact, and moved toward workflow integration. Overall, a unified synthesis through a health-systems lens is needed to map the distribution of evidence, characterize methodological choices, and identify translational gaps that constrain real-world adoption.

To address these gaps, we focus on five guiding questions: **(RQ1)** How are AI approaches distributed across the surgical pathway, including their targeted clinical tasks and the data modalities used? **(RQ2)** What are the characteristics of the datasets underpinning these studies, including their geographic origins, sample sizes, and accessibility? **(RQ3)** What modeling approaches and training strategies have been adopted, and what evidence is reported to support generalizability, including external validation? **(RQ4)** How is model performance assessed and reported, and how far have these systems advanced toward workflow integration and real-world deployment? **(RQ5)** How have research trends evolved over time, including global participation and institutional contributions, and what do they suggest about the maturity of the field?

To answer these questions, this scoping review provides a comprehensive and structured examination of AI-enabled decision support, primarily deep learning-based systems, across the epilepsy surgery pathway. We first summarize the clinical context of existing studies, including their surgical stages, targeted clinical tasks, and data modalities. We then characterize the underlying datasets by assessing their geographic sources, sample sizes, and accessibility. Next, we synthesize modeling approaches and training strategies, with particular attention to evidence supporting generalizability, including external validation practices. We also examine how performance is assessed and reported, and how far systems have advanced toward workflow integration and real-world deployment. Finally, we review publication patterns and institutional contributions to contextualize the evolution of the field. Together, these analyses map the current landscape, clarify implementation-relevant evidence gaps, and highlight priorities for developing and evaluating AI systems that can be adopted in real-world care pathways and across centers.

## Results

### Overview of the Scope

**Figure 1:**
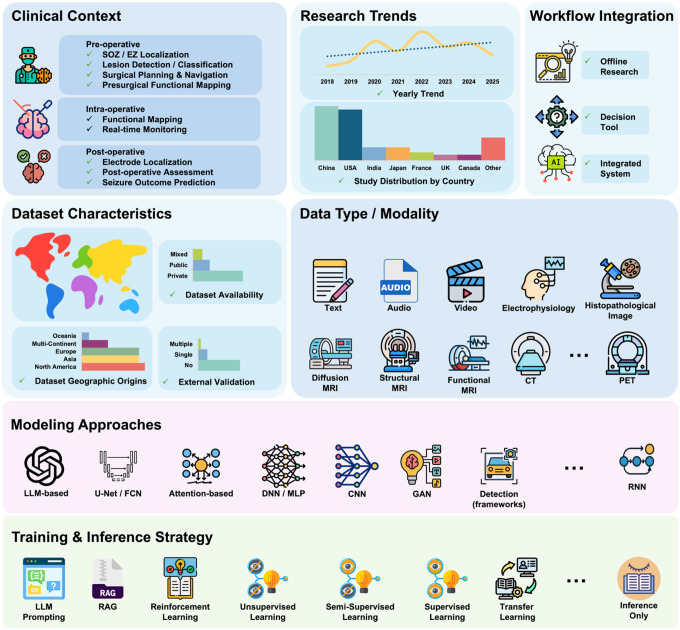
Overview of AI-enabled decision support systems in epilepsy surgery and the key analytical dimensions of this scoping review. The figure summarizes how the included studies were organized across the epilepsy surgery pathway, including surgical stages and clinical tasks, dataset characteristics and data modalities, modeling approaches and training strategies, and levels of workflow integration, together with publication trends used to synthesize the evidence base. Icons sourced from Flaticon.com (full attributions in Supplementary Note 1).

This section provides an overview of the scoping review and summarizes its principal findings (Fig. 1). We synthesize the literature on AI-enabled decision support in epilepsy surgery across five implementation-relevant dimensions: clinical context along the surgical pathway, dataset characteristics, modeling approaches and evidence supporting generalizability, clinical integration, and research trends.

**Figure 2:**
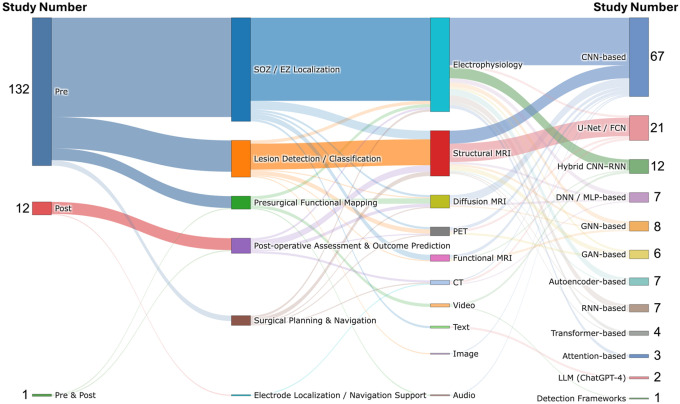
Sankey diagram of AI-enabled decision support studies in epilepsy surgery. Flows map included studies from surgical stage to target clinical task, data modality, and model architecture. Link width is proportional to the number of studies along each pathway.

**Figure 3:**
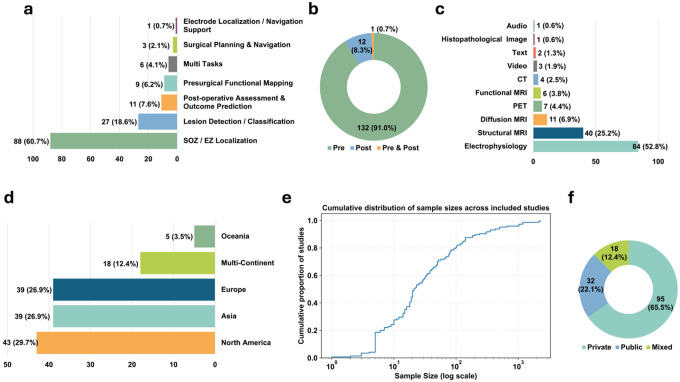
Clinical contexts and dataset characteristics of included studies in epilepsy surgery. (a) Targeted clinical tasks. (b) Surgical stage coverage (pre-operative, post-operative, or both). (c) Data modalities used. (d) Geographic provenance of datasets. (e) Cumulative distribution of sample sizes across studies (log scale). (f) Dataset accessibility (private, public, or mixed).

**Figure 4:**
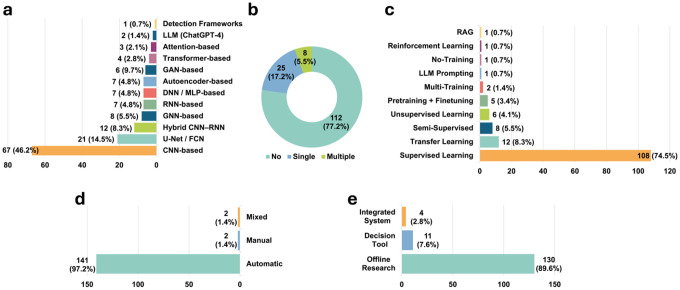
Methodological and implementation-relevant characteristics of included studies in epilepsy surgery. (a) Model architecture categories. (b) External validation (none, single-center, or multi-center). (c) Training strategies. (d) Evaluation approach (automatic, manual, or mixed). (e) Workflow integration stages (offline research, decision-support tool, or integrated system).

**Figure 5:**
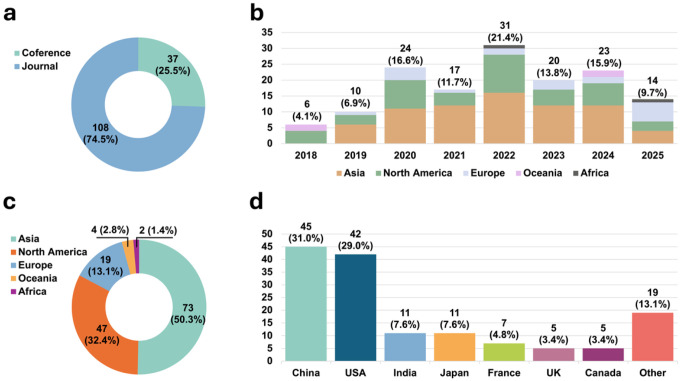
Publication landscape of included AI-enabled decision support studies in epilepsy surgery (2018–2025). (a) Publication types (journal article, or conference paper). (b) Annual publication trends by region. (c) Regional distribution. (d) Country distribution.

As illustrated in Fig. 2 and Fig. 3, we first map AI applications across the surgical pathway by characterizing targeted clinical tasks and associated data modalities **(RQ1)**. We then examine the datasets used in these studies (Fig. 3), including their geographic origins, sample sizes, and levels of accessibility **(RQ2)**. Next, we synthesize modeling approaches and training strategies (Fig. 4), with attention to evidence reported to support generalizability, including external validation **(RQ3)**. We also review how performance is assessed and reported, and how far systems have advanced toward workflow integration and real-world deployment (Fig. 4; **RQ4**). Finally, in Fig. 5, we contextualize the evolution of the field by summarizing publication trends, study types, and regional contributions **(RQ5)**. Collectively, these results provide a pathway-level view of where evidence is concentrated and where implementation-relevant gaps remain, helping to prioritize future prospective evaluation and workflow integration efforts for adoption across centers.

### Study Selection and Characteristics

Fig. 7 summarizes the study selection process. Of the 2,534 records initially identified, 1,299 duplicates were removed, leaving 1,235 records for title and abstract screening. At this stage, 854 records were excluded for not meeting the predefined inclusion criteria, resulting in 381 reports eligible for full-text review. During full-text screening, 236 records were further excluded due to the following reasons: out-of-scope setting or context (N=5), ineligible study design (N=54), or irrelevant methods (N=177). Ultimately, 145 studies were included in this scoping review.

Across the included studies, evidence was heavily concentrated in the pre-operative stage (91%, N=132), with relatively few post-operative studies (8.3%, N=12), and one study spanning both stages. No included studies specifically addressed the intra-operative stage, highlighting a clear gap in pathway coverage. Within the pre-operative stage, the most common tasks were SOZ/EZ localization (60.7%, N=88), and lesion detection/classification (18.6%, N=27), followed by presurgical functional mapping (6.2%, N=9) and surgical planning and navigation (2.1%, N=3). Post-operative studies primarily focused on post-operative assessment and outcome prediction (7.6%, N=11), with one study addressing electrode localization/navigation support (0.7%, N=1). Data modalities varied by task, with electrophysiology data (scalp EEG, intracranial EEG, and MEG) commonly used for SOZ/EZ localization, and structural MRI predominantly used for lesion detection and classification. Dataset provenance showed broad geographic coverage, with 29.7% (N=43) using data from North America, 26.9% (N=39) from Asia, 26.9% (N=39) from Europe, and 3.5% (N=5) from Oceania. Multi-regional datasets were used in 12.4% of studies (N=18), reflecting an emerging trend toward broader data diversity. Modeling approaches were dominated by CNN-based methods (46.2%, N=67), followed by U-Net/FCN-based segmentation models (14.5%, N=21), with smaller proportions of hybrid CNN–RNN models (8.3%, N=12) and GNN-based architectures (5.5%, N=8). Two studies (1.4%) used large language models (LLMs), specifically ChatGPT-4, reflecting early exploration of foundation model approaches in this domain. Additional LLM-based studies^34,35^ identified during screening were excluded because they focused on surgical candidate selection or pre-surgical triage rather than tasks within the epilepsy surgery pathway, consistent with our predefined criteria. Implementation readiness remained limited. Most studies were conducted as offline research (89.6%, N=130), a smaller proportion functioned as decision-support tools (7.6%, N=11), and only four studies (2.8%) were integrated into clinical systems. Publication output consisted of journal articles (74.5%, N=108) and conference papers (25.5%, N=37), with activity increasing over time, including notable increases in 2020 (16.6%, N=24) and 2022 (21.4%, N=31).

### Clinical Context

As illustrated in Fig. 3a–c, we summarize the clinical contexts of the included studies, including their targeted clinical tasks, surgical stages, and data modalities. Among these studies, 91% (N=132) focused on the pre-operative stage, with the main clinical tasks involving SOZ/EZ localization, lesion detection/classification, presurgical functional mapping, and surgical planning and navigation. Notably, 60.7% (N=88) of all included studies centered on SOZ/EZ localization, making it the predominant research focus in current deep learning applications for epilepsy surgery. For example, recent studies have leveraged various electrophysiology modalities, including scalp EEG^23,36–43^, intracranial EEG^9–11,44–69^, and MEG^70–77^, to identify seizure-onset and epileptogenic zones. Other works have utilized MRI based data, such as structural MRI^78–80^ and resting-state fMRI^81–85^, and two studies have analyzed clinical text^21,86^ for SOZ/EZ localization. Beyond single modalities, several works also incorporated multimodal inputs. For instance, Li et al.^87^ and Yang et al.^88^ combined intracranial EEG and scalp EEG for SOZ/EZ localization. In addition, multiple studies such as Jeong et al.^89^, Fard et al.^90^, Banerjee et al.^91^, and Zotova et al.^92^ employed various imaging modalities to perform SOZ/EZ localization, including MRI (T1, T2, FLAIR, and DWI) and PET. A total of 27 studies (18.6%) focused on lesion detection/classification task, most of which relied on structural MRI with multiple sequences^6–8,24,93–108^, Intracranial EEG^109,110^, pathology^111^, or multimodal inputs^112–114^ for lesion detection/classification. Nine studies (6.2%) focused on presurgical functional mapping, and commonly used data modalities included diffusion MRI tractography^13,115–117^, motion visual analysis^12,118,119^, intracranial EEG^120^, and audio speech^121^. The remaining three studies (2.1%) focused on surgical planning and navigation. Granados et al.^14^ proposed an early-fusion multimodal approach integrating T1-weighted MRI, DWI, and CT. Nejedly et al.^16^ introduced a late-fusion multimodal method based on intracranial EEG and T1-weighted MRI. In contrast, Liu et al.^15^ relied solely on T1-weighted MRI to perform surgical planning and navigation. Unlike the single-task studies summarized above, five studies addressed multiple pre-operative tasks. Hossain et al.^122^ and Zhang et al.^123^ used scalp EEG and intracranial EEG, respectively, to perform both SOZ/EZ localization and presurgical functional mapping. Mo et al.^124^ employed an early-fusion multimodal approach integrating MRI sequences with T1, FLAIR and PET to achieve lesion detection/classification and surgical planning and navigation. Similarly, Park et al.^125^ applied an early-fusion multimodal strategy, based on T1-weighted MRI and T2-FLAIR, to perform SOZ/EZ localization and lesion detection/classification. In addition, Hu et al.^126^ used scalp EEG for both SOZ/EZ localization and surgical planning and navigation.

In contrast, 8.3% (N=12) of studies addressed the post-operative stage, primarily targeting post-operative assessment and outcome prediction^17–20,22,127–132^ and electrode localization/navigation support^133^. A variety of data modalities were applied in this stage, including diffusion tractography MRI^20,127^, structural MRI^22,128,129,131,132^, CT^130,133^, and electrophysiology (Intracranial EEG)^17,19^. Notably, Tang et al.^18^ employed a multimodal late-fusion approach that integrated multi-sequence MRI (T1, T2, and FLAIR), CT, and PET to achieve post-operative assessment and outcome prediction. Additionally, one study examined both stages^134^, covering presurgical functional mapping in the pre-operative phase and postoperative assessment and outcome prediction in the post-operative phase.

Overall, current AI-enabled decision-support research in epilepsy surgery remains heavily concentrated in the pre-operative stage, with SOZ/EZ localization and structural MRI–based lesion detection/classification accounting for most studies. In contrast, presurgical functional mapping, surgical planning and navigation, and post-operative evaluation remain relatively underrepresented, and no study in our review specifically addressed intra-operative applications. Although multimodal fusion has begun to appear in recent work, its adoption is still limited, and comprehensive multi-task models remain uncommon. The absence of intra-operative studies also indicates limited evidence for real-time, workflow-integrated decision support during surgery.

### Data Characteristics

Across the included studies, 10 different data modalities and types were used. Electrophysiology was the most common (52.8%, N=84), primarily consisting of intracranial EEG^9–11,44–69^, scalp EEG^23,36–43^, and MEG^70–77^. This was followed by structural MRI (25.2%, N=40), mainly including T1-weighted, T2-weighted, and FLAIR MRI; diffusion MRI (6.9%, N=11), including DWI^13,14,20,89,91,115,116,134^, DTI^127^, and DKI^108^; PET^90,124^ (4.4%, N=7); functional MRI (rs-fMRI)^81,84^ (3.8%, N=6); and CT^133^ (2.5%, N=4). One study use histopathology for FCD classification^111^. Seven studies incorporated video^12,118,119^, text^21,86^, or audio data^121^. Notably, many studies did not rely on a single modality for epilepsy surgery analysis. Instead, they employed combinations of multiple data modalities, such as the fusion of T2-weighted and FLAIR MRI^98,99^, intracranial EEG with rs-fMRI^112^, intracranial EEG with scalp EEG^88^, and the integration of multiple MRI sequences with PET^114,124,135^ or CT^14,18^. Collectively, the breadth of modalities and heterogeneous acquisition protocols highlight the need for data harmonization and interoperable pipelines to support cross-center deployment.

Based on the geographic distribution of the datasets used in the included studies (Fig. 3d), most data originated from North America (29.7%, N=43), Asia (26.9%, N=39), and Europe (26.9%, N=39), with Oceania contributing 3.5% (N=5). In addition, 12.4% (N=18) of the studies utilized multi-regional datasets spanning two or more continents. For example, Yang et al.^88^ combined intracranial EEG and scalp EEG data from Asia and Europe for pre-operative SOZ/EZ localization. Zhang et al.^135^ similarly used data from Asia and Europe, but employed T1-weighted MRI, FLAIR MRI, and PET with a multimodal early-fusion strategy to achieve lesion detection/classification. Gill et al.^7^ also adopted an early-fusion multimodal approach for lesion detection/classification, using T1-weighted MRI and FLAIR MRI collected from four regions: Asia, Europe, North America, and South America. The studies by Spitzer et al.^104^ and Ripart et al.^106^ incorporated the broadest geographic coverage to support lesion detection/classification, with T1-weighted and FLAIR MRI sourced from five regions (Asia, Europe, Oceania, North America, and South America). In contrast to the imaging- and electrophysiology-based studies above, Luo et al.^86^ utilized clinical text collected from Asia, Europe, and North America to perform SOZ/EZ localization. Overall, the predominance of single-region datasets suggests that geographic representativeness and cross-center generalizability remain important constraints for implementation at scale.

Most studies relied on private datasets (65.5%, N=95), spanning diverse data types including audio, video, text, CT, PET, and multiple MRI sequences. In contrast, 22.1% (N=32) used fully public datasets, of which 30 involved electrophysiology data, including scalp EEG and intracranial EEG, and two studies used structural MRI (T1-weighted and FLAIR). Another 12.4% (N=18) employed mixed datasets combining private and public sources, mainly covering text, electrophysiology, PET, and T1-weighted MRI. The dataset sizes exhibited substantial heterogeneity, ranging from single-subject datasets to large cohorts exceeding 2,000 patients. The cumulative distribution (Fig. 3e) shows a strongly right-skewed, long-tailed pattern, with 80% of the studies using fewer than 100 patients. Only a small proportion of studies^40,78,87,95,111,135^ used medium-sized datasets (100–500 patients), and very few utilized large-scale cohorts^81,96,104^ (>1,000 patients). Collectively, the dataset landscape is characterized by strong reliance on small, private, single-region datasets, with limited public data availability, which can impede external validation, reproducibility, and workflow-integrated evaluation needed for adoption across centers.

### Modeling Approaches

To support AI-enabled decision support across diverse modalities, the included studies adopted a range of deep learning architectures (Fig. 4a). CNN-based models were the most common (46.2%, N=67), spanning 1D CNNs for electrophysiology^17,41,51,110,115,136^, 2D CNNs for MRI^84,85^, and 3D CNN for volumetric MRI^81,96,114,124^. In addition, 14.5% (N=21) used CNN-derived segmentation architectures, primarily U-Net or fully convolutional networks (FCNs), applied to PET^113,135^, CT^130,133^, and structural MRI data^8,15,22,95,98,100,102^. A further 8.3% (N=12) and 4.8% (N=7) of the studies adopted RNN-based^137–139^ and hybrid CNN–RNN^140,141^ architectures, respectively, most often for electrophysiology or video analysis. The remaining studies used a variety of model architectures, such as autoencoder-based^142,143^, Transformer-based^144^, and attention-based^68,70^ models for specific tasks. Notably, one study^119^ leveraged an existing detection framework (Mask R-CNN) to perform automated presurgical functional mapping from video data, and two studies^21,86^ applied large language models (LLMs; ChatGPT-4) to clinical text for SOZ/EZ localization. For example, Luo et al.^86^ addressed the SOZ/EZ localization task by using ChatGPT-4, which interpreted textual seizure semiology descriptions from literature and clinical records to automatically predict the most likely brain lobe of seizure onset.

The training strategies were dominated by supervised learning, which accounted for 74.5% (N=108) of all models (Fig. 4c). Transfer learning was used in 8.3% (N=12) of the studies, typically through initialization with pretrained CNN backbones^18,37,118^. A smaller proportion employed semi-supervised learning^145–147^ (5.5%, N=8) or unsupervised learning techniques^143,148^ (4.1%, N=6), such as autoencoder-based feature learning^143^. Only a limited number of studies utilized more advanced training paradigms, such as pretraining followed by finetuning^56,69,74,75,129^ (3.4%, N=5), multi-training schemes^9,117^ (1.4%, N=2), or reinforcement learning^149^ (0.7%, N=1). A small number of studies required no additional training^119^ (0.7%, N=1) or relied purely on LLM prompting^86^ (0.7%, N=1). One study additionally incorporated retrieval-augmented generation (RAG) for text-based clinical reasoning^21^ (0.7%, N=1). Overall, these patterns suggest that most systems depend on labeled data and task-specific training, which may limit scalability when high-quality annotations are difficult to obtain.

Evidence supporting generalizability remained limited (Fig. 4b). Most studies did not perform external validation (77.2%, N=112). A smaller subset conducted single-center external validation (17.2%, N=25), typically using an independent cohort from a different institution within the same geographic region. Several studies^23,57,65,86,88,104,135,145^ validated models on external data from different geographic regions, providing a stronger test of cross-regional generalizability. Only 5.5% (N=8) of the studies^7,38,74,106,124,129,132,141^ employed multi-center external validation, leveraging datasets from two or more independent sites to assess model generalizability across institutions. Taken together, while the field explores diverse architectures and training strategies, current epilepsy surgery AI is still dominated by supervised CNN-based approaches, and the scarcity of multi-center external validation indicates substantial gaps in implementation-relevant evidence for deployment across centers.

### Evaluation and Clinical Integration

As shown in Fig. 4d, most studies assessed model performance using fully automatic evaluation procedures (97.2%, N=141), in which quantitative performance metrics were computed directly from algorithmic outputs without additional human involvement. Common metrics included classification measures such as accuracy, sensitivity, specificity, AUC, F1-score^40,78,84,144^, as well as segmentation measures such as IoU and DSC^7,106,135^. Only 1.4% (N=2) of the studies relied exclusively on manual evaluation. For example, Chiang et al.^21^ developed an ontology-guided, GPT-based system to assist SOZ/EZ Localization from clinical text, and epilepsy specialists manually judged each model prediction as correct or incorrect to derive accuracy. Similarly, Pastore et al.^107^ manually verified their algorithm generated lesion predictions against histopathology or scalp EEG findings. Another 1.4% (N=2) of studies employed mixed evaluation, combining automated quantitative metrics with expert review to adjudicate ambiguous cases or assess clinical plausibility. For instance, in Mo et al.^124^, sensitivity, specificity, and accuracy were automatically computed, but overlap validation and all trajectory feasibility and safety assessments were manually determined by clinical experts.

With respect to clinical integration (Fig. 4e), most systems remained offline research prototypes (89.6%, N=130), without integration into routine clinical software environments or workflows. A smaller subset (7.6%, N=11) functioned as standalone decision-support tools^18,22,23,38,39,73,74,84,106,111,124^, in which clinicians accessed model outputs through independent research interfaces operating outside routine clinical systems such as PACS, SEEG analysis software, or platforms. In contrast, only 2.8% (N=4) of the studies reported semi-integrated clinical workflows, in which AI outputs were incorporated directly into interfaces already used in clinical practice, such as PACS viewers^107^, online SEEG review platforms^69^, or GUI-based electrophysiology analysis tools^150^, and prospectively validated MRI-based lesion detection systems applied in daily clinical reading^24^. These systems enabled clinicians to visualize, review, and interact with model predictions within existing workflows, but none were fully automated or embedded into hospital information systems.

Taken together, performance evaluation remains predominantly algorithm-centric and automated, while evidence for workflow-integrated use is limited. Most systems remain offline research tools, and only a small minority report partial, clinician-in-the-loop integration, highlighting ongoing gaps in implementation readiness for deployment across centers.

### Publication Trends

This section characterizes publication trends in epilepsy surgery AI research (Fig. 5). Overall, publication activity has increased substantially since 2018, with a notable rise between 2020 and 2022. Most studies were published as journals articles (74.5%, N=108), while conferences papers accounted for 25.5% (N=37) (Fig. 5a). Regionally, Asia and North America contributed the largest shares of publications (50.3%, N=73 and 32.4%, N=47, respectively). Followed by Europe (13.1%, N=19), Oceania (2.8%, N=4), and Africa (1.4%, N=2). At the country level, China produced the most studies (31.0%, N=45), followed by the United States (29.0%, N=42). India and Japan each contributed 7.6% (N=11), while France, the United Kingdom, and Canada contributed 4.8% (N=7), 3.4% (N=5), and 3.4% (N=5), respectively. The remaining countries collectively contributed 13.1% (N=19) of publications. Overall, these patterns indicate growing research activity and broadening international participation, while also highlighting uneven geographic distribution of evidence generation.

## Discussion

Our results highlight substantial gaps in pathway coverage and implementation readiness for AI-enabled decision support in epilepsy surgery. Evidence is heavily concentrated in the pre-operative stage (91%), with only 8.3% of studies addressing post-operative evaluation and none specifically targeting intra-operative applications. This imbalance reflects differences in data accessibility, operational constraints, and clinical risk. For example, pre-operative EEG and MRI are routinely acquired with established protocols, whereas intra-operative data collection is technically challenging, time-sensitive, and often constrained by surgical workflow. Beyond stage imbalance, effective seizure focus localization often requires integrating structural imaging, electrophysiology, and seizure semiology. However, most studies focus on either imaging (e.g., MRI) or electrophysiology (EEG) in isolation, and comparatively few integrate these modalities with the video-based behavioral and semiology information. Meanwhile, intra-operative AI remains substantially underdeveloped despite its clear clinical importance. Future research should prioritize evidence generation for intra-operative, workflow-integrated decision support^120^, and develop pathway-level approaches that connect pre-operative, intra-operative, and post-operative stages through end-to-end or multi-stage pipelines. Within pre-operative studies, SOZ/EZ localization and lesion detection/classification dominate current research activity. These tasks often have relatively well-defined reference standards (e.g., SEEG-confirmed seizure onset zones, postoperative seizure outcomes), making them easier to benchmark. In contrast, tasks such as presurgical functional mapping and surgical planning/navigation more closely reflect real clinical decision making but remain relatively underexplored. This imbalance in task coverage may constrain clinical impact by leaving critical components of the care pathway insufficiently supported. Although multimodal analyses can offer advantages over single-modality approaches, few studies conduct systematic comparisons of how different fusion strategies, including early^7,14,87,100,101^, late^16,18,112^, or intermediate^113,151^ fusion, affect performance and interpretability. Advancing this field will require standardized and interoperable multimodal pipelines, along with interpretable cross-modal alignment methods that integrate electrophysiology, structural and functional imaging, and clinical information into unified decision-support systems.

Generalizability and implementation readiness are constrained by the current data landscape. Across included studies, dataset size represents a major limitation. 80% of studies used small cohorts with fewer than 100 patients, while large-scale datasets (>1,000 patients) were extremely rare. This reflects the inherent difficulty of collecting large, high-quality epilepsy surgery datasets, particularly those involving multimodal imaging or invasive electrophysiology^23,92,126^. However, reliance on small datasets increases the risk of overfitting and limits the reliability of model performance estimates^12,44,76^. To address these challenges, future research should leverage advanced methods such as self-supervised learning, semi-supervised learning, and foundation models to enhance representation learning and improve generalization under small dataset conditions. Geographic and institutional coverage is similarly uneven. Most datasets originate from North America, Asia, and Europe, and are predominantly single-center and not publicly available. Such single-region, single-center datasets may embed systematic biases related to patient demographics, scanner types, acquisition protocols, and surgical practices, thereby reducing the applicability of trained models to other populations or institutions. Moving forward, increased cross-regional collaboration and harmonization techniques such as domain adaptation and federated learning will be important to mitigate these biases^89,141^. Data accessibility represents an additional challenge. With 65.5% of datasets being private and unavailable for reuse, reproducibility is often limited, and fair method comparison remains challenging. Establishing open, multi-center, multimodal epilepsy surgery benchmarks, alongside broader sharing of pretrained models, and code, will be critical for accelerating progress and improving community confidence^68,93,129^. Beyond scale and accessibility, label quality also presents inherent limitations^145^. Ground-truth labels such as SOZ/EZ localization, FCD lesion masks, or HFO annotations frequently contain noise due to subjective expert interpretation, heterogeneous clinical standards, or reliance on proxy markers such as resection zones or short-term postoperative outcomes. Improved multi-rater consensus labeling, uncertainty modeling, and the use of long-term outcomes will be needed to strengthen the reliability of training and evaluation.

Finally, data modality usage remains highly skewed. Electrophysiology modalities account for 52.8% of all datasets, whereas other rich information sources, such as PET, rs-fMRI, diffusion imaging, and especially clinical text, are rarely exploited. With recent advances in large language models, leveraging clinical narratives, reports, and semiology descriptions may open new opportunities for improving prediction accuracy and capturing patient-specific clinical context. Expanding beyond electrophysiology to more diverse data modalities will be an important direction for future work^21,118^.

In terms of modeling, the field remains dominated by supervised CNN-based approaches. Most studies directly adapt existing architectures from general computer vision or signal processing, rather than models specifically tailored to the characteristics of multimodal neuroimaging, electrophysiology, or surgical decision-making. Although these models often produce strong results on small, single-center datasets, their robustness in multi-center, heterogeneous clinical environments remains largely unknown^6,106^. Moreover, there remains a notable absence of systematic comparisons across model families, such as CNNs, transformers, GNNs, hybrid architectures, or generative models, making it difficult to determine which design choices consistently translate across settings. Notably, large language models (LLMs) have recently been introduced into this field, primarily for interpreting clinical text for SOZ/EZ localization^21,86^. However, their application remains limited to unimodal textual reasoning. Future work should explore the potential of multimodal LLMs as decision-support agents capable of integrating imaging, electrophysiology, and clinical narratives. LLMs may also support auxiliary tasks, including EEG/MRI annotation, study design, and automated report generation. A common limitation is the lack of external validation. With 77.2% of studies reporting no external validation, performance estimates are likely inflated due to single-center bias. Strengthening cross-center and cross-region generalization should therefore be a central methodological priority. Multi-center external validation, domain adaptation, and harmonization strategies will be essential to ensure that models perform reliably across diverse patient populations and clinical environments^76,111^.

Evaluation and translation evidence remain key bottlenecks for deploying epilepsy surgery AI in health systems. Most studies rely solely on automated quantitative metrics such as accuracy, AUC, or Dice scores. While these measures are essential for algorithmic benchmarking, they provide limited insight into whether a model improves clinically meaningful outcomes in epilepsy surgery. Incorporating clinically relevant endpoints, such as changes in surgical planning, accuracy of resection margin prediction, seizure-freedom rates, reduction in evaluation time, or improvements in clinician confidence, would provide a more comprehensive assessment of real-world utility^22,84^. Workflow integration is also uncommon. 89.6% of included studies operate as offline research prototypes, with only a small number developed into decision-support tools or semi-integrated systems. This translation gap reflects a broader disconnect between algorithm development and real clinical workflows, driven by challenges such as limited access to surgical environments, high barriers to software integration, compatibility issues with existing medical devices and IT infrastructure, unclear regulatory requirements, and the lack of collaboration between researchers, clinical engineers, and industry partners^80,84,120^. Moving forward, evaluations should measure real clinical impact rather than just accuracy, including effects on surgical planning, workflow efficiency, and clinician confidence. Furthermore, AI systems must be usable in real workflows, support clinician-in-the-loop interaction, and integrate smoothly with existing systems^73^.

Publication volume in epilepsy surgery AI, predominantly deep learning-based, has increased since 2018 with notable accelerations in 2020 and 2022. This growth likely reflects the maturation of deep learning frameworks, increased availability of open-source tools and datasets, and broader adoption of computational methods within the neuroscience and medical imaging communities. Despite this momentum, the field still lacks standardized benchmarks or shared evaluation pipelines, which limits reproducibility and makes it difficult to compare methodological advances across studies. The geographic distribution of contributing research groups is similarly imbalanced. Asia and North America account for most publications, while contributions from Europe are moderate and those from other regions remain limited. This imbalance raises concerns regarding model fairness and global applicability, as datasets predominantly represent specific populations, acquisition protocols, and surgical practices^86^. Addressing these disparities will require coordinated multi-center consortia, greater international data sharing, and wider adoption of federated learning to support collaborative model development without requiring data exchange. These efforts are essential for building globally representative datasets and developing more generalizable and equitable AI-enabled decision-support systems for epilepsy surgery.

**Figure 6:**
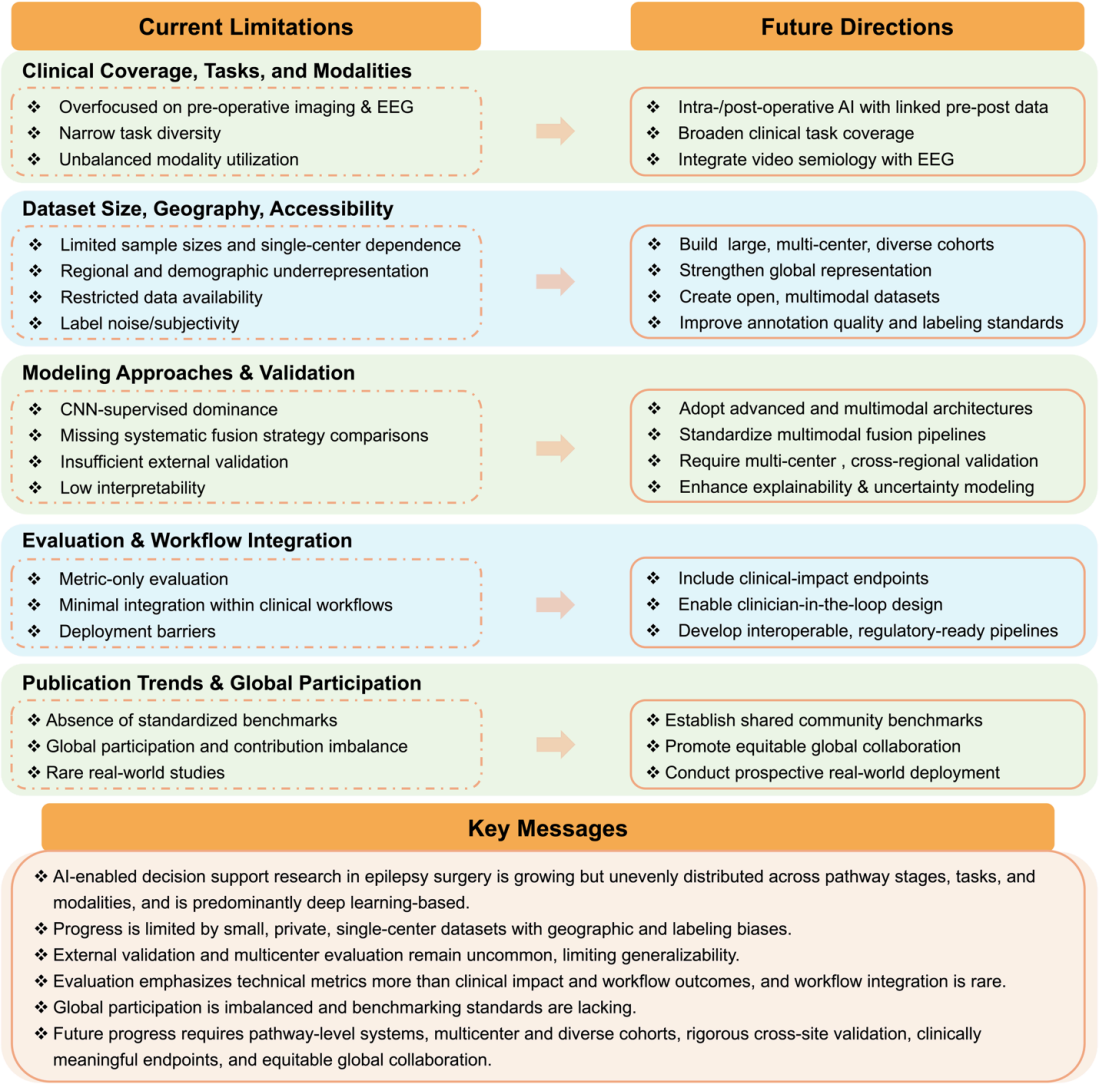
Summary of limitations, future directions, and key take-home messages for AI-enabled decision support systems in epilepsy surgery. The figure summarizes major gaps in pathway coverage, data resources, modeling and validation, evaluation and translation, and global participation, and highlights corresponding research priorities to support safe, scalable, and equitable adoption.

Taken together, our findings indicate that epilepsy surgery AI is expanding but remains fragmented across the care pathway. Evidence is constrained by small, geographically skewed, and often non-public datasets, alongside limited external validation and scarce multi-center evaluation. Most models perform well in controlled research settings but remain untested in the heterogeneous, multi-center environments where real clinical decisions are made. Evaluation practices emphasize technical accuracy rather than clinical impact, and workflow-integrated deployment is uncommon. Addressing these gaps will require a transition toward full-pathway AI systems, large-scale multimodal datasets, advanced and interpretable modeling strategies, rigorous generalization testing, and stronger collaboration across technical, clinical, and industry domains. Fig. 6 summarizes these limitations and outlines future directions for developing reliable, equitable, and clinically actionable decision-support systems for epilepsy surgery.

This survey also has several limitations. First, we restricted our search to English language publications, which may have excluded relevant studies reported in other languages. Second, our review focused on peer-reviewed articles and did not incorporate recent preprints, potentially missing emerging research trends. In summary, this scoping review provides a comprehensive and structured synthesis of AI-enabled decision support in epilepsy surgery. We examined how models are distributed across clinical stages and tasks, characterized the underlying datasets, analyzed modeling approaches and validation strategies, and assessed evaluation practices and workflow integration. We further identified key methodological, data-related, and translational limitations and outlined future directions needed to advance this field. Our findings highlight implementation-relevant gaps, including uneven pathway coverage, small and geographically concentrated datasets, limited external validation, and scarce workflow-integrated evaluation. We hope this work serves as a foundational reference for clinicians, researchers, and industry partners seeking to develop reliable, generalizable, and clinically impactful decision-support systems for epilepsy surgery.

## Methods

### Data Source and Article Selection

**Figure 7:**
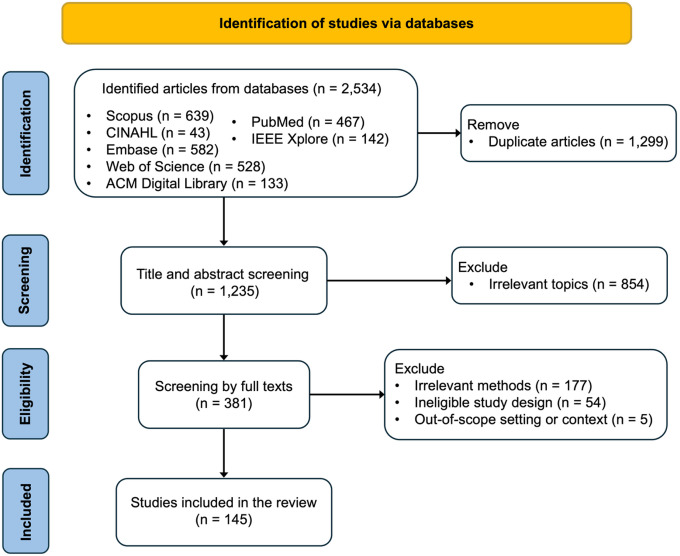
PRISMA-ScR flowchart of study selection process. The flowchart summarizes the numbers of records identified from databases, duplicates removed, records screened, full-text articles assessed for eligibility, studies included, and the main reasons for exclusion at each stage.

This scoping review was reported in accordance with the PRISMA-ScR guidelines, and the study selection process is summarized in the PRISMA flow diagram (Fig. 7). The protocol for this scoping review was not prospectively registered. A systematic search was conducted across seven electronic databases, including Scopus, Web of Science, Embase, PubMed, IEEE Xplore, ACM Digital Library, and CINAHL, to identify studies on AI-enabled decision support in epilepsy surgery published between January 2018 and May 2025. The search strategy combined terms related to deep learning, epilepsy, and surgery. Specifically, search terms related to deep learning were “deep learning”, “machine learning”, “neural network*”, “artificial intelligence”, “AI”, “LLM*”, “large language model*”, “foundation model*”, “vision language model*”, “vision-language model”; search terms related to epilepsy were “epilep*”, and “seizure*”; and search terms related to surgery involved “surgery”, “surgical”, “presurgical”, “preoperative”, “intraoperative”, “postoperative”. Detailed search queries and Boolean operators (AND/OR) for each database are provided in the Supplementary Table 1. Study selection followed a two-stage screening process. First, two reviewers independently screened titles and abstracts using predefined inclusion and exclusion criteria. Second, full texts of potentially eligible studies were independently evaluated, with disagreements resolved through discussion or, if necessary, adjudication by a third reviewer. Specifically, studies were included if they met the following criteria: (1) empirical investigations of deep learning-based methods; (2) applications directly related to epilepsy surgery, including pre-operative, intra-operative, or post-operative stages. Conversely, studies were excluded if they: (1) relied solely on traditional machine learning methods; (2) focused on epilepsy-related applications that were not connected to the surgical pathway; (3) solely for surgical candidate screening or pre-surgical triage without involving tasks directly related to the operative workflow, or (4) were non-English publications, review articles, editorials, or abstracts without accessible full text.

### Data Extraction and Analysis

Data from the included studies were systematically extracted using a predefined template to capture key information (Supplementary Table 2). Extracted items included: (1) basic study information, including title, publication type, published year, and author region (based on the first author’s primary institutional affiliation: Asia, Europe, North America, Oceania, or Africa); (2) epilepsy surgery stage & clinical task, specifying the targeted surgical stage (pre-operative, intra-operative, or post-operative), and the corresponding clinical task; (3) data characteristics, including data source region, data modality, data size, and dataset accessibility (public, private, or mixed). In this survey, data size was recorded as the number of patients whose data were used in the study, including both individuals with epilepsy and healthy controls; (4) modeling details, including deep model architecture and training strategy (e.g., supervised learning, transfer learning, self-supervised learning, weak supervision, federated learning, or other specified strategies); (5) evaluation, including external validation, evaluation type (manual, automatic, or mixed), reported evaluation metrics, and the primary performance summary; and (6) clinical translation, in which we categorized the deployment level of each deep learning system as offline research (models evaluated retrospectively without clinical use), standalone decision support tools (outputs available for clinician review but not embedded), or semi-integrated clinical systems (outputs partially incorporated into clinical workflows). All data extraction was performed independently by two reviewers, with discrepancies resolved through discussion and consensus.

The basic information, clinical context, dataset characteristics, methodological details, and translational relevance of the included deep learning studies were systematically documented and categorized using our taxonomy. We synthesized these data to highlight key patterns in AI-enabled decision support systems for epilepsy surgery. We first summarized the survey scope, including surgery stage, clinical tasks, data modalities, and model architectures. We then analyzed study metadata such as geographic distribution, data accessibility, and training strategies. Finally, we reviewed evaluation practices and clinical translation stages and outlined major limitations and future directions.

## Supplementary Material

Supplementary Files

This is a list of supplementary files associated with this preprint. Click to download.

- AIenableddecisionsupportsystemsinepilepsysurgeryascopingreviewsupplementary.pdf

## Data Availability

All data generated during this study are presented in the Supplementary Materials.

## Box 1 | Terms and Concepts

### Large Language Models (LLMs):

Large-scale neural networks trained on massive text corpora to acquire general language understanding and reasoning capabilities. LLMs can interpret clinical narratives, summarize reports, assist decision-making, and serve as generalizable backbones for medical tasks.

### LLM Prompting:

Providing tailored instructions, demonstrations, or constraints to an LLM to guide its output toward a target task without fine-tuning. Includes zero-shot, few-shot, and chain-of-thought.

### Retrieval-Augmented Generation (RAG):

A framework that combines external knowledge retrieval with generative modeling, enabling models to produce responses that are accurate, up-to-date, and grounded in verifiable sources.

### Foundation Models (FMs):

Large-scale pre-trained models that learn generalizable representations across modalities or tasks, providing adaptable backbones for downstream medical applications.

### Transfer Learning:

Fine-tuning models pre-trained on large datasets (e.g., ImageNet MRI models) for epilepsy-specific tasks.

### Self-supervised Learning (SSL):

Training models using proxy tasks (e.g., contrastive learning, masked modeling) to learn robust representations from unlabeled neuroimaging or EEG data.

### Semi-supervised Learning:

Training with both labeled and unlabeled data, often using pseudolabeling or consistency regularization to mitigate label scarcity.

### Unsupervised Learning:

Learning patterns without explicit labels—for example anomaly detection, clustering, or autoencoder feature representation.

### Reinforcement Learning (RL):

Learning decision policies from reward signals, occasionally applied to trajectory planning or electrode optimization.

### Fusion Strategies:

Definitions of early, intermediate, late, and hybrid fusion, describing how multimodal data are integrated.

### External Validation:

Testing model generalizability on independent datasets from different centers or regions, categorized as single-center or multi-center external validation.

## Code Availability

No code was generated during this study.
